# Cost minimization analysis of indication-specific osteosynthesis material in oral and maxillofacial surgery

**DOI:** 10.1007/s10006-022-01126-2

**Published:** 2022-11-04

**Authors:** Adrian Franke, Bernhard Weiland, Michaela Bučkova, Christian Bräuer, Günter Lauer, Henry Leonhardt

**Affiliations:** 1 Department of Oral and Maxillofacial Surgery, University Hospital Carl Gustav Carus, 01304 Dresden, Germany; 2 Klinik und Poliklinik für Mund-, Kiefer- und Gesichtschirurgie, Universitätsklinikum Carl Gustav Carus, an der Technischen Universität Dresden, 01304 Dresden, Germany

**Keywords:** Maxillofacial surgery, Time, Costs, Prepackaged, Documentation time, EU regulation

## Abstract

**Purpose:**

Following the introduction of the Regulation (EU) 2017/745 by the European Parliament, any bioactive substance or surgical implant introduced into the human body must be documented. The regulation requires any implant to be traced back to the manufacturer. Lot numbers need to be available for every single medical implant. Also, the manufacturer is required by law to provide implants individually packaged and sterilized. Previously, model tray systems (MOS tray) were used for osteosynthesis in oral and maxillofacial surgery, in which the individual implants could not be registered separately. The new regulation made it impossible to use such processes during surgery anymore and a need for a change in the medical practice surged. We examined a possible solution for the new legislation. The aim of this prospective cohort study is to analyze the MOS tray systems to osteosynthesis materials prepackaged in sets. We record and evaluate parameters such as surgical time and documentation time. We perform a short cost analysis of our clinic. The primary aim is to determine how much time is gained or lost by the mandatory increased patient safety. The secondary aim is to describe change in costs.

**Methods:**

Patients that underwent standard surgical procedures in the clinic of oral and maxillofacial surgery of the faculty hospital Carl Gustav Carus in Dresden were included. We chose open reduction and internal fixation (ORIF) of anterior mandibular corpus fractures as well as mandibular advancement by means of bilateral sagittal split osteotomies (BSSO) as standardized procedures. Both of these procedures require two osteosynthesis plates and at least four screws for each plate. MOS trays were compared to prepackaged sterilized sets. The sets include a drill bit, two plates, and eight 5-mm screws.

A total number of 40 patients were examined. We allocated 20 patients to the ORIF group and the other 20 patients to the BSSO group. Each group was evenly subdivided into a MOS tray group and a prepackaged group. Parameters such as the incision-suture time (IST) as well as the documentation time (DT) by the operating room (OR) staff to complete documentation for the implants are the main focus of investigation.

**Results:**

For open reduction, the incision-suture time was significantly different in favor of the MOS tray (*p* < 0.05). There was no difference in the BSSO groups. However, we observed a significantly different (*p* < 0.01) documentation time advantage for the prepackaged sets in both the ORIF and BSSO groups. On top of that, we find that by using the prepackaged kits, we are able to reduce sterilization costs by €11.53 per size-reduced container. Also, there is also a total cut of costs of €38.90 and €43.70, respectively, per standardized procedure for implant material.

**Conclusions:**

By law, a change in the method of approaching surgery is necessary. For standardized procedures, the right choice of implants can lead to a reduction of documentation time and costs for implant material, sterilization, as well as utilizing less instruments. This in turn leads to lower costs for perioperative processing as well as provision of state-of-the-art implant quality implementing higher patient security.

## Introduction

On 5 April 2017, the European Parliament published the Regulation (EU) 2017/745 that any bioactive substance or surgical implant introduced into the human body must be documented [1]. Its date of implementation was 26 May 2021. The reason for these measures were defective implants used for breast augmentation, which caused silicone leakage into the tissue [2]. There was an urgent need for the legislation to react [3]. The regulation stipulates that any implant introduced into the human body must be able to be traced back to the manufacturer. Thus, any implant needs its own batch number and requires a medical device passport to be handed to every patient that receives such a medical implant.

The manufacturers are obliged by law to provide implants that are individually packaged and sterilized. In some scenarios, i.e., for standardized procedures, there is also a possibility to provide individually packaged implantation sets. Up to now, miniplate osteosynthesis system trays (MOS tray) were used in which the implants could not be registered separately and the new regulation made it impossible to use accompanying processes during surgery anymore. In order to increase the patient’s safety and track the plates as well as the screws, individually packaged osteosynthesis material with batch numbers has been introduced. These prepackaged sets of osteosynthesis material with individually packed plates and screws cause a change of process during surgery.

## Objective

The aim of this prospective cohort study is to analyze and compare the processes connected to osteosynthesis using either the MOS tray systems or the osteosynthesis materials prepackaged in sets. Time for surgery and the time for documentation were recorded and evaluated. In addition, the costs involved were assessed. The primary aim is to determine how much time is gained or lost by the mandatory increased patient safety. The secondary aim is to describe change in costs.

## Methods

This prospective open study included patients that received surgical treatment for lower jaw fractures or mandibular osteotomies following standard surgical procedures at the Department of Oral and Maxillofacial Surgery of the University Hospital Carl Gustav Carus in Dresden. Inclusion criteria were open reduction and internal fixation (ORIF) of simple, non-comminuted anterior mandibular fractures or orthognathic surgery by means of bilateral sagittal split osteotomies (BSSO) with mandibular advancement and subsequent plate osteosynthesis. Usually, in both procedures, two osteosynthesis plates and four screws for each plate are required. Exclusion criteria were patients of minor age, patients that were not able to give written consent, and, in the case of anterior mandibular fractures, edentulous jaws. When more than two osteosynthesis plates or more than a total of eight screws were used, patients were excluded from this study.

**Fig. 1:**
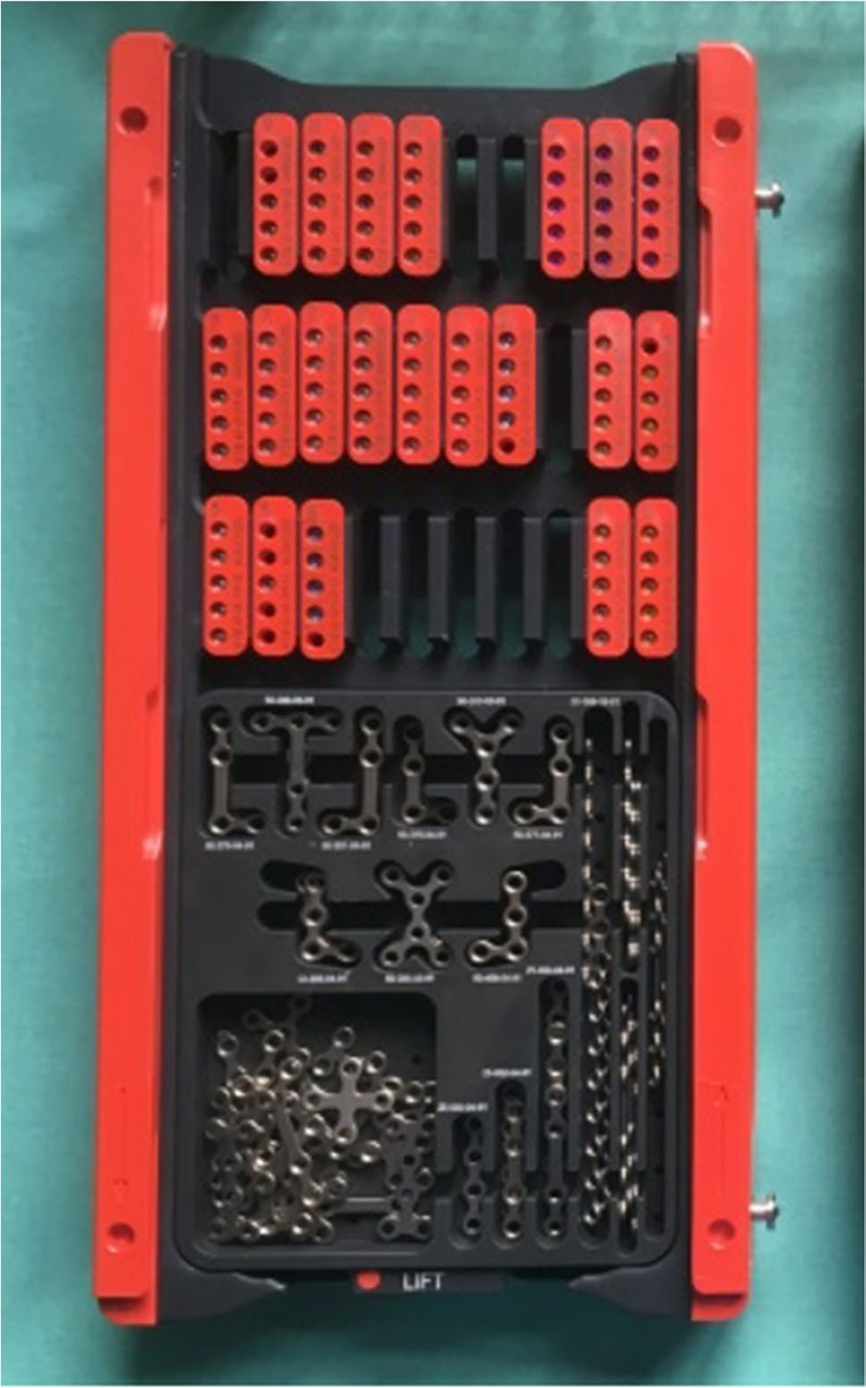
KLS Martin model tray system; photo: A. Franke, UKD

**Fig. 2:**
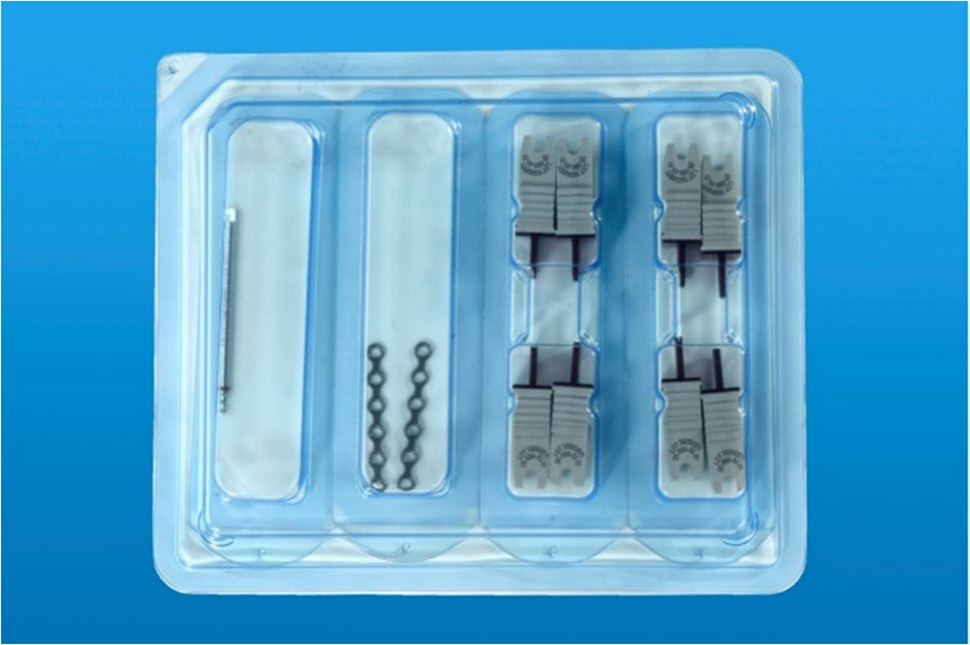
KLS Martin prepackaged set, example for BSSO; Photo: G. Bellmann, UKD

A MOS tray (Fig. 1) contains different types of osteosynthesis plates and small racks for screws in various lengths as well as drill bits in several lengths. These trays get refilled and resterilized and are being used for both types of surgery. There are two different prepackaged sterilized sets (Sterile kit Mini). The sets for ORIF contain a drill bit (Twist drill with J-notch attachment, 1.5 × 5 mm), two four-hole plates (LevelOne Fixation Osteosynthesis 2.0 Mini, standard profile), and eight Mini screws (2.0 × 5 mm, MaxDrive®), the sets for BSSO two six-hole instead of the four-hole plates (Fig. 2). All the sets were provided by KLS Martin GmbH & Co. KG.

A total number of 40 procedures in 40 patients were assessed. In 20 patients, the ORIF of anterior mandibular fractures was analyzed. In 20 patients, the BSSO was evaluated. In each group, we used the MOS tray for ten patients and the prepackaged sets for the other ten patients.

Parameters such as the incision-suture time (IST) as well as the time documentation for documentation for the implants (DT) were assessed.

The prices for the materials were provided by KLS Martin GmbH & Co. KG upon request. Costs for the osteosynthesis materials were compared between the price for the prepackaged kits and the sum of the single items used in the MOS tray. Also the costs for sterilization of containers was analyzed and taken into consideration. All prices in this study are presented in Euroes (€).

For clinical documentation, we used the program ORBIS version 3.7 from Agfa Healthcare N.V. We obtained the statistical evaluation from PAST (version 4.10). We computed descriptive statistics and tested for normal distribution by Kolmogorov–Smirnov test. When even distribution was confirmed, Welch’s unequal variances t-test was used for comparison of the two groups, where *p* < 0.05 was considered significant.

## Results

### ORIF of anterior mandibular fractures

**Fig. 3:**
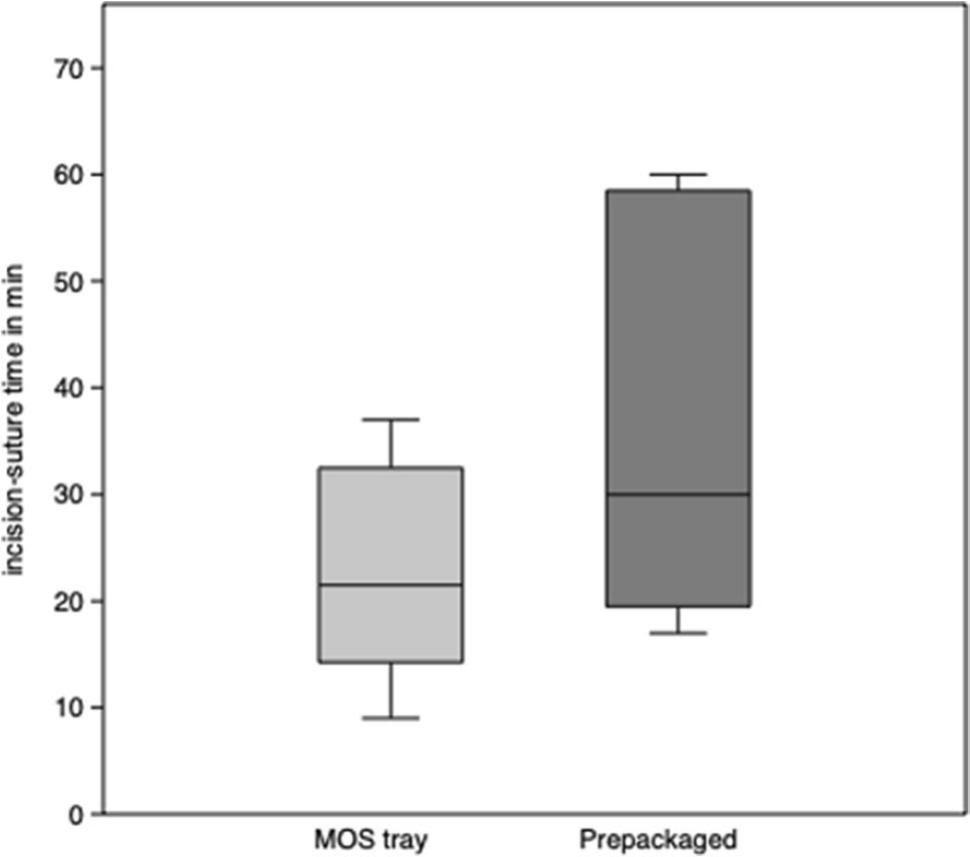
Incision-suture time (IST) for paramedian mandibular fractures. Box plots depict the interquartile range, the line shows the median, and the whiskers represent the minimum and maximum values recorded

The incision-suture times for the MOS tray systems range from 9 to 37 min with a mean of 22.5 ± 9.7 min (median 21.5 min) and for the prepackaged sets from 17 to 60 min with a mean of 35.2 ± 17.7 min (median 30 min). There is a significant difference (*p* < 0.05, 95% confidence interval − 26.4 to − 0.2) between the MOS tray system and the prepackaged sets (Fig. 3).

**Fig. 4:**
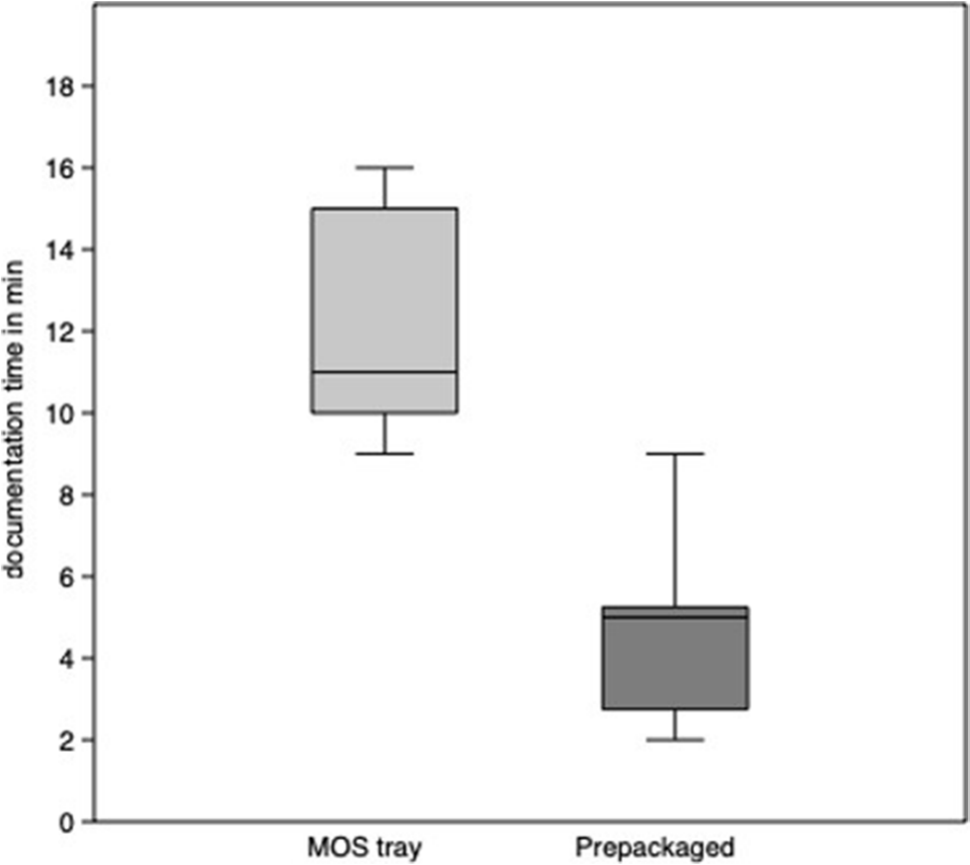
Documentation time (DT) for anterior mandibular fractures. Box plots depict the interquartile range, the line shows the median, and the whiskers represent the minimum and maximum values recorded

The time of documentation for the MOS tray systems ranges from 9 to 16 min with an average of 12.1 ± 2.6 min (median 13.5 min) and for the prepackaged sets from 2 to 9 min with an average of 4.6 ± 2.1 min (median 5.5 min). Thus, the DT is significantly shorter (*p* < 0.001; 95% confidence interval 5.3 to 9.7) for prepackaged sets (Fig. 4).

#### BSSO

**Fig. 5:**
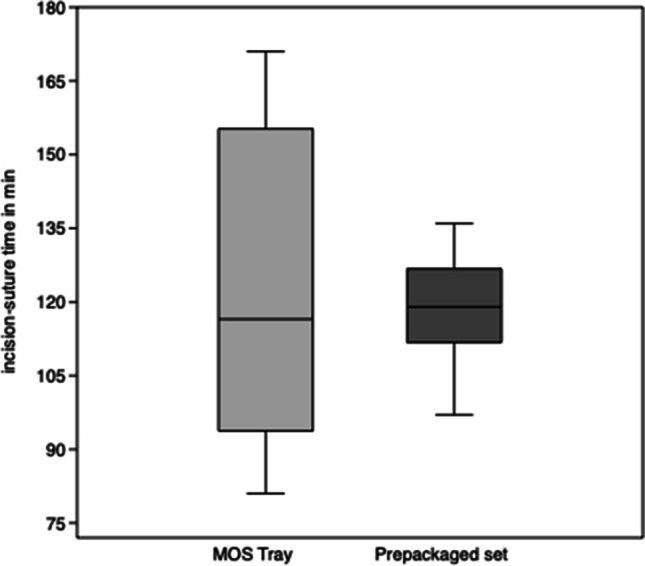
Incision-suture time (IST) for mandibular advancement (BSSO). Box plots depict the interquartile range, the line shows the median, and the whiskers represent the minimum and maximum values recorded

In orthodontic procedures using the MOS tray system, ISTs lasted from 81 to 171 min with a mean of 122.4 ± 32.5 min (median 116.5 min) and using the prepackaged sets from 97 to 136 min with an average of 118.1 ± 11.0 min (median 119 min). There were no significant differences (*p* = 0.69; 95% confidence interval − 19.6 to 28.2) for IST between the two groups (Fig. 5).

**Fig. 6:**
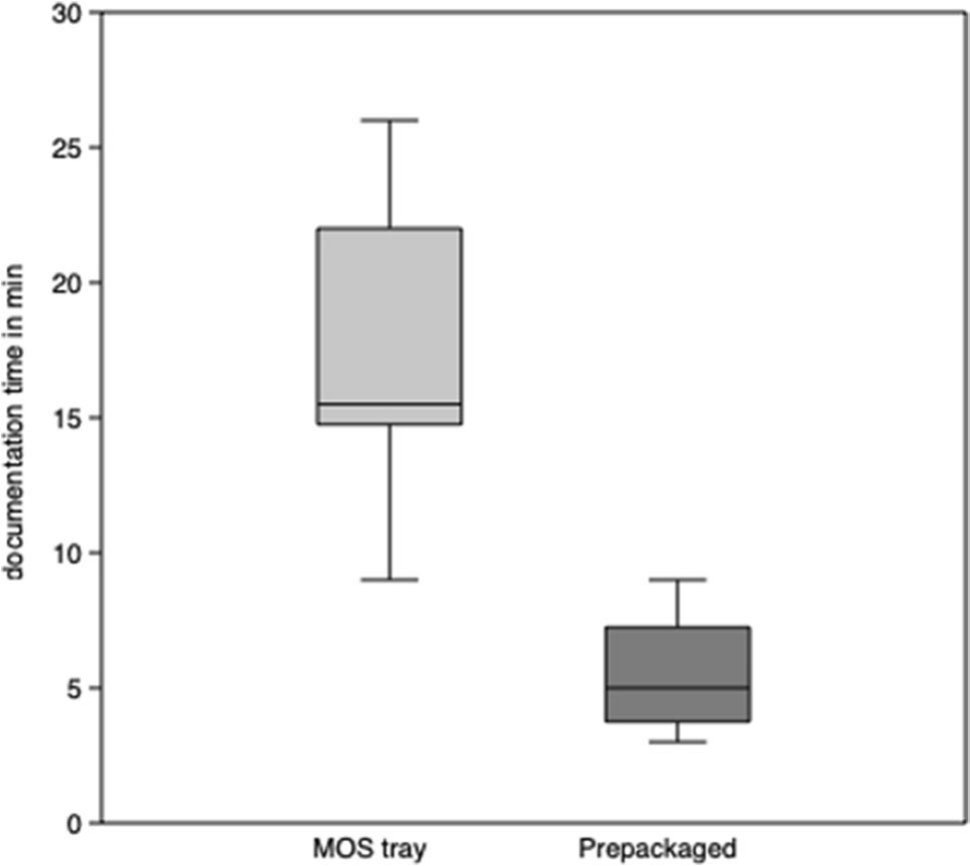
Documentation time (DT) for mandibular advancement (BSSO). Box plots depict the interquartile range, the line shows the median, and the whiskers represent the minimum and maximum values recorded

For the MOS tray systems, DTs lasted between 9 and 26 min with an average of 17.5 ± 5.3 min (median 15 min) and for the prepackaged sets between 3 and 9 min with an average of 5.4 ± 2.1 min (median 5 min). The results reflect a significant time advantage (*p* < 0.001; 95% confidence interval 6.8 to 14.6) (Fig. 6).

### Costs

Using the prepackaged sets, we are able to reduce the size of the containers for instruments that need to be sterilized by half. This means that we can reduce the costs of sterilization for every container from €32.19 to €20.66 in our facility when using prepackaged sets.

Costs for implant material when using the MOS tray to treat a standard anterior mandibular fracture are as follows: for a single four-hole plate €45.10, for a single MaxDrive screw €18.10, and for a drill-bit €73.40 adding to €308.40. A single prepackaged set for this procedure costs a total of €270.50. Thus, costs of €38.90 are saved when using prepackaged sets in anterior mandibular fractures.

The costs for osteosynthesis of a BSSO using the MOS tray are as follows: for a single six-hole plate €48.00, for a single MaxDrive screw €18.10, and for a drill-bit €73.40 adding to €314.20. The price for a prepackaged set costs a total of €270.50. Costs of osteosynthesis material for a BSSO are cut by €43.70 when using prepackaged sets.

## Discussion

### Incision-suture times

In ORIF of anterior mandibular fractures, there was a significant difference between the MOS tray and prepackaged set groups in favor of the MOS tray. Although even simple uncomminuted fractures were chosen, the specific complexity or degree of displacement of fractures was not taken into account. Thus, treating mandibular fractures may have to be regarded as non-standardized procedures. Another aspect is the fact that with increased experience of the surgeon, there is also a swifter approach to the surgical procedure. For the aforementioned procedures, inexperienced as well as experienced surgeons of our clinic performed the surgery. The reason for the discrepancy we found, however, cannot to be explained.

A recent study reports surgery times of 52 to 86 min for treatment of anterior mandibular fractures [4]. Another study’s results show surgery times of 37 to 47 min [5]. This concludes that the results of our study are very acceptable for both of the subgroups in ORIF of anterior mandibular fractures.

Current literature reports surgery times for BSSO to average between 80 and 126 min [6–8]. The BSSO performed by us can be considered a standardized procedure as our results are similar to the ones reported in literature. On average, there are no time advantages in the MOS tray group as compared to the prepackaged set group. However, it is interesting to compare the ranges of the two subgroups. The MOS tray subgroup shows a significantly higher spread of ISTs. The reason for this may again lay in the surgeon’s experience performing the operation.

In summary, there is no time advantage in IST for using either of the systems in both ORIF and BSSO.

### Documentation time

There are several reports covering documentation times for surgery [9, 10]. These deal with the implementation of an electronic health record (EHR). Interestingly, a key factor is the time needed for documentation. In our study, we found a significant reduction of DT for the prepackaged sets in both the ORIF in anterior mandibular fractures as well as the BSSO group. This is of superior importance in times of rising requirements of documenting more and more details around patients and their treatment. Not only is it helping to increase patient’s safety, but it is also required by law [1]. We found the new process to ease documentation as well. Using the MOS tray, every single implant and screw had to be typed in by hand into the surgery protocol. The prepackaged set contains several parts and only has a single lot number. By means of a QR code, the documentation process is extremely simplified. Thus, qualified personnel is liberated for other tasks.

### Costs

The use of prepackaged sets eliminates the need to clean and sterilize an entire MOS tray. For the central sterilization unit in the university hospital Dresden, this leads to reduction of sterilization costs of €11.53 per size-reduced container applied for the prepackaged sets. Other advantages of smaller containers are reduced weight, easier packing, faster sterilization time, as well as highly reduced time of checking every single implant within the MOS tray. Yet again, qualified personnel is liberated for other tasks. In literature there are similar results by utilizing less space in the sterilization process [11–14]. Besides reducing costs for sterilization, less instruments in a container in turn also mean less weight of the container for handling and less time to set up the surgery nurse’s table, which in return also reduces preparation time for surgery [15]. In our study, this time advantage is not reflected in IST or DT.

One should also not neglect the lower price of €38.90 respectively €43.70 per prepackaged set. This is especially true, when there is a high number of standardized procedures performed in a clinic. Unused implants in the MOS trays are sterilized several times, sometimes over the course of months or even years. This leads to alteration of the alloy composition as well as the microstructure of the implants [16]. Nowadays, when patient security is of paramount importance, this is not acceptable anymore.

### Limitations

Limitations of the study are that there may be different prices for osteosynthesis materials depending on special contracts for various institutes. Hence, it may be that the data cannot be extrapolated for every clinic. The same is true for sterilization costs that may vary from clinic to clinic. In general, the cost data should be transferable for the practicing surgeon.

Time intervals for surgery and documentation highly depend on the skill and experience of the surgeon, the assisting surgeon, and the supporting OR personnel. These factors are difficult to estimate and, thus, may influence the results of this study.

## Conclusion

It is apparent that a change in the method of approaching surgery is necessary. With the experience of the surgeon and careful preoperative planning, the choice of implants can be made preoperatively with a high level of certainty for standardized procedures, which may result in reduction of documentation time, perioperative time and costs for sterilization and osteosynthesis materials as well as utilizing less instruments. This in turn leads to lower costs for perioperative processing as well as provision of state-of-the-art implant quality implementing higher patient security. We conclude the change in method to be economically feasible.

## Data Availability

The datasets generated and/or analyzed during the study are available from the corresponding author on reasonable request.
